# Plasma thrombomodulin is a valuable biomarker to predict the severity of hemorrhagic fever with renal syndrome caused by the Hantaan virus

**DOI:** 10.3389/fcimb.2025.1563621

**Published:** 2025-04-28

**Authors:** Han-Dong Zhao, Yan Zhang, Xiao-Hong Wang, Hong-Bo Qian, Tong-Bo Yu, Peng Li, Kang-Xiao Ma, Hong-Li Liu

**Affiliations:** ^1^ Central Laboratory of Virology, Shaanxi Provincial Hospital of Infectious Diseases, The Xi’an Eighth Hospital, Xi’an, China; ^2^ Clinical Laboratory Center, Shaanxi Provincial Hospital of Infectious Diseases, The Xi’an Eighth Hospital, Xi’an, China; ^3^ Experimental Center of Xi’an Academy of Traditional Chinese Medicine, Xi’an Hospital of Traditional Chinese Medicine, Xi’an Affiliated Hospital of Shaanxi University of Chinese Medicine, Xi’an, China; ^4^ Shaanxi Provincial Clinical Medical Research Center of Infectious Diseases, Shaanxi Provincial Hospital of Infectious Diseases, The Xi’an Eighth Hospital, Xi’an, China; ^5^ Clinical Laboratory Center, Xi’an People’s Hospital (Xi’an Fourth Hospital), Affiliated People’s Hospital of Northwest University, Xi’an, China

**Keywords:** HFRS, Hantaan virus, thrombomodulin, disease monitoring, evaluation

## Abstract

**Objectives:**

This study aimed to investigate the Thrombomodulin (TM) levels in patients who suffered hemorrhagic fever with renal syndrome (HFRS) of varying severities, and to evaluate the predictive properties of TM for the seriousness of HFRS, thereby providing a clue for the monitoring and management of this patients in the future.

**Methods:**

Chemiluminescence was used to determine the concentrations of TM in 196 patients with HFRS and 49 healthy controls. Conventional testing techniques were used to test the basic clinical reference values for leukocytes, platelets (PLT), C-reactive protein (CRP), creatine (Cr), uric acid (UA), and urea, and the values for activated partial thromboplastin time, prothrombin time, and fibrinogen. The colloidal gold method was used to measure HFRS antibody levels in the patients. The correlation of TM with conventional parameters was assessed using Spearman correlation analysis, and ordinal logistic regression analysis was used to analyze the severity risk factors. The predictive potency of TM for HFRS patients’ severity was evaluated by receiver operating characteristics (ROC) curve analysis.

**Results:**

The concentrations of TM increased with disease severity and peaked in the critical type patients. In addition, plasma levels of TM were proportionally correlated with the levels of leukocytes (*r*= 0.4218; *p*<0.01), creatine (*r*= 0.3797; *p*<0.01), urea (*r*= 0.3763; *p*<0.01), uric acid (*r*= 0.3624; *p*<0.01), and C-reactive protein (*r*= 0.2767; *p*<0.01). Conversely, there was an inverse correlation between TM, platelet counts (*r*= -0.4509; *p*<0.01), and fibrinogen levels (*r*= -0.2431; *p*<0.01). Furthermore, TM demonstrated significant predictive value for the severity of HFRS with an area under the ROC curve (AUC) of 0.872(95% *CI*: 0.822-0.921, *P*<0.001).

**Conclusions:**

TM levels are associated with HFRS severity, suggesting that TM detection might be beneficial for monitoring the status and effective management of HFRS patients.

## 1 Introduction

Hemorrhagic fever with renal syndrome (HFRS) is a zoonotic disease that seriously threatens public health and socio-economic around the world. It can be caused by the Hantaan virus (HTNV), a single-stranded negative RNA virus that belongs to the Hantaviridae family under the Bunyavirales Order (Schmaljohn et al., 1985; Walker et al., 2019). Rodents have been demonstrated to be the main natural hosts for HFRS-causing hantaviruses, although other species, including bats, moles, shrews, reptiles, and fish, were found to carry hantaviruses (Vial et al., 2023). People can be infected by inhaling virus-contaminated aerosols from saliva, urine, and feces of infected rodents (Avsic-Zupanc et al., 2019; Vaheri et al., 2013a). The most common signs of HFRS are fever, bleeding, renal impairment, and thrombocytopenia, furthermore, comorbidities affecting the nephrological, cardiovascular, and endocrinological systems can also be found after HTNV infection (Muranyi et al., 2005; Vaheri et al., 2013b). HFRS is widely prevalent in Europe and East Asia, and China is the most affected country since 60,000-150,000 cases are reported annually worldwide, 90% of which are registered in this region (Sehgal et al., 2023; Yan et al., 2007). Additionally, the National Health Commission of the People’s Republic of China reported that from 1950 to 2014, 1,625,002 HFRS cases and 46,968 HFRS-related deaths were confirmed in China, resulting in a mortality rate of 2.89% (Jiang et al., 2016). Noteworthy, with the increasing attention paid to the pathogenesis of HFRS, it has been suggested that endothelial cells of capillaries and small vessels are the principal targets of hantaviruses, and increased vascular permeability is central to the pathological changes in HFRS (Mertz et al., 1998; Avsic-Zupanc et al., 2019). So far, considerable efforts have been made to elucidate the pathogenic mechanism of HFRS, effective support treatment, and vaccination greatly contribute to the containment of HTNV infection. However, a reliable clinical parameter for improving the management of HFRS patients, which could be used as clinical references for severity assessment and prognosis prediction is urgently needed.

Thrombomodulin (TM), a type 1 transmembrane glycoprotein, was discovered in the 1980s and is considered central to crucial biological processes and biochemical pathways such as the regulation of coagulation, innate immunity, inflammation, and cell proliferation (Esmon and Owen, 2004; Loghmani and Conway, 2018). Notably, TM can be expressed by various cell types including capillary endothelial cells of the alveolar zones of the lungs, syncytiotrophoblasts of the human placenta, endothelium of all blood vessels (arteries, veins, and capillaries), and lymphatic endothelial cells, suggesting multiple roles of this molecule and close associations with various diseases to some extent (Maruyama et al., 1985; Kawanami et al., 2000). Indeed, early studies have documented that TM possesses tumor-suppressor capacity, dampening cell proliferation, invasion, and metastasis (Hosaka et al., 2000; Chang et al., 2016). Meanwhile, it was found that the level of TM in endothelial cells that overly atherosclerotic lesions were down-regulated, consistent with local increases in thrombosis and inflammation (Laszik et al., 2001). Additionally, TM was reported to be central in protecting the heart, lungs, and kidneys from ischemia-reperfusion. A close relationship between TM and diabetic nephropathy, and pulmonary disease has also been demonstrated (Loghmani and Conway, 2018). However, the association between TM expression and the severity of HFRS was rarely reported. Since the central role of TM in the regulation of coagulation, innate immunity, and inflammation as well as the fact that the pathogenesis of HFRS is documented to be related to immune dysfunction and pathological damage caused by the dysregulation of immunity coupled with the consensus that the endothelium is closely associated with HTNV infection (Kraus et al., 2004). Thus, we explored the potential link between TM level change and the course of HFRS.

Overall, in this study, we investigated the expression of TM in patients with HFRS of varying phases; analyzed the correlation of TM with the parameters of renal function, coagulation, and inflammation; and assessed the correspondence between changes in TM levels and HFRS severity. Furthermore, the predictive property of TM for the seriousness of HFRS was evaluated using receiver operating characteristics (ROC) curve analysis.

## 2 Materials and methods

### 2.1 Participants

A total of 245 individuals aged 10–78 years were enrolled in this study. Of these, 196 were patients, and 49 were healthy donors. The recruited patients were treated at the Department of Infectious Diseases of Shaanxi Provincial Hospital of Infectious Diseases (The Xi’an Eighth Hospital) from September 2022 to March 2024. The recruitment strategy was based on the HFRS criteria for clinical diagnosis and classification, established by the National Health Commission of China (China, 2008). In summary, patients were included if they were positive for HFRS serological test coupled with a history of travel or living in an epidemic area, or have a record of direct or indirect interaction with rodent excreta with the subsequent manifestation of fever, fatigue, nausea, vomiting, bleeding, hypotensive shock, or kidney injury. Exclusion criteria included other infections similar to HFRS, kidney, cardiovascular, hematological, autoimmune, viral hepatitis, or other liver diseases, and lack of clinical data. The medical records, general clinical information, and laboratory examination results were recorded in detail. All data were analyzed anonymously and informed consent was obtained from the participants. All procedures followed ethical standards outlined in the 1964 Helsinki Declaration and its subsequent amendments or comparable ethical standards and were approved by the Institutional Review Board of Shaanxi Provincial Hospital of Infectious Disease (No. 20220618).

### 2.2 Procedures and definitions

The severity of HFRS patients was classified into four types according to the HFRS criteria of clinical classification: mild group, patients suffered kidney injury without obvious hypotension and oliguria; moderate group, patients suffered hypotension, skin and mucous membrane bleeding, bulbar conjunctiva, acute kidney injury (AKI) along with typical oliguria; severe group, patients suffering severe uremia, hypotension, skin and mucous membrane bleeding, bulbar conjunctiva and either peritoneal or pleural effusion, and AKI with urine output of 50–500 mL/day for ≤ 5 days or urine output of < 100 mL/day for ≤ 2 days; critical group, patients suffering one or more of the following complications: refractory shock (≥ 2 days), severe AKI with urine output of 50–500 mL/day for > 5 days or urine output of < 100 mL/day for > 2 days, severe secondary infection, or heart failure in addition to the clinical characteristics commonly observed in severe cases, visceral hemorrhage, pulmonary and brain edema.

### 2.3 Detection of conventional laboratory indices

Venous blood samples were collected from each patient and healthy donor population. Following collection, the samples were centrifuged at 2000 rpm for 10 min at 4 °C within 2 h of the blood collection. Nine laboratory parameters were routinely detected by automated analyzers (Mindray SAL9000, Mindray BC6900, or Mindray ExC810; Mindray Medical, China), including white blood cells (WBC), platelets (PLT), activated partial thromboplastin time (APTT), prothrombin time (PT), fibrinogen, creatinine (Cr), urea, uric acid (UA), and C-reactive protein (CRP).

### 2.4 Detection of HTNV antibody

The titers of antibodies specific to HTNV were determined using the colloidal gold method (Bosheng Biotechnology, China). This involved mixing a sample diluent (100 µL) with a plasma sample anticoagulated with EDTA-K_2_ (2 µL). The resulting mixture was pipetted onto a test board (70 µL) and incubated for 20 minutes, according to the manufacturer’s instructions. The sensitivity and specificity of the kits were 96.71% and 98.72%, respectively.

### 2.5 Detection of TM

Venous blood was drawn from the HFRS patients and healthy donors and collected into a sodium citrate anticoagulation tube, followed by incubation at 37 °C for ten minutes. Centrifugation was then conducted to separate the plasma at 3000 rpm for ten minutes. TM levels were determined by the Chemiluminescence immunoassay (CLIA) method with an automatic analyzer according to the manufacturer’s instructions (Wondfo I2900, Wondfo Bio, China). Briefly, 10 µL plasma coupled with 50 µL magnetic bead working solution was added to the reaction cup, and incubated at 37 °C for five minutes followed by washing, then 50 µL alkaline phosphatase (ALP) labeled working solution was added, and incubated at 37 °C for five minutes before the luminescence signals that released by the double-antibody sandwich method complex (antibody-coated magnetic beads-TM-ALP labeled antibody) were measured by the analyzer at the 470 nm wavelength. The examination range of the kit was 0–200 TU/mL without dilution.

### 2.6 Statistical analysis

Statistical analysis was conducted using GraphPad Prism 8.0 software (GraphPad Software, LLC, USA). Tables and figures were created using Excel 2021 (Microsoft Corporation, USA) and Origin 2021 software (OriginLab Corporation, USA). Non-normally distributed variables are shown as medians with interquartile ranges (IQR) and the data were analyzed using the Mann-Whitney U test or Kruskal-Wallis H test. Spearman correlation analysis was performed to assess the correlation between TM levels and conventional laboratory indices. The severity risk factor was analyzed by ordinal logistic regression analysis, and the receiver operating characteristics (ROC) curve was used to evaluate the predictive capacity of TM for the severity of HFRS and was qualified by the area under the ROC curve (AUC) and the 95% confidence interval (CI). A two-sided *P* value of less than 0.05 was considered statistically significant.

## 3 Results

### 3.1 Clinical typing and demographic characteristics of HFRS patients

**Figure 1:**
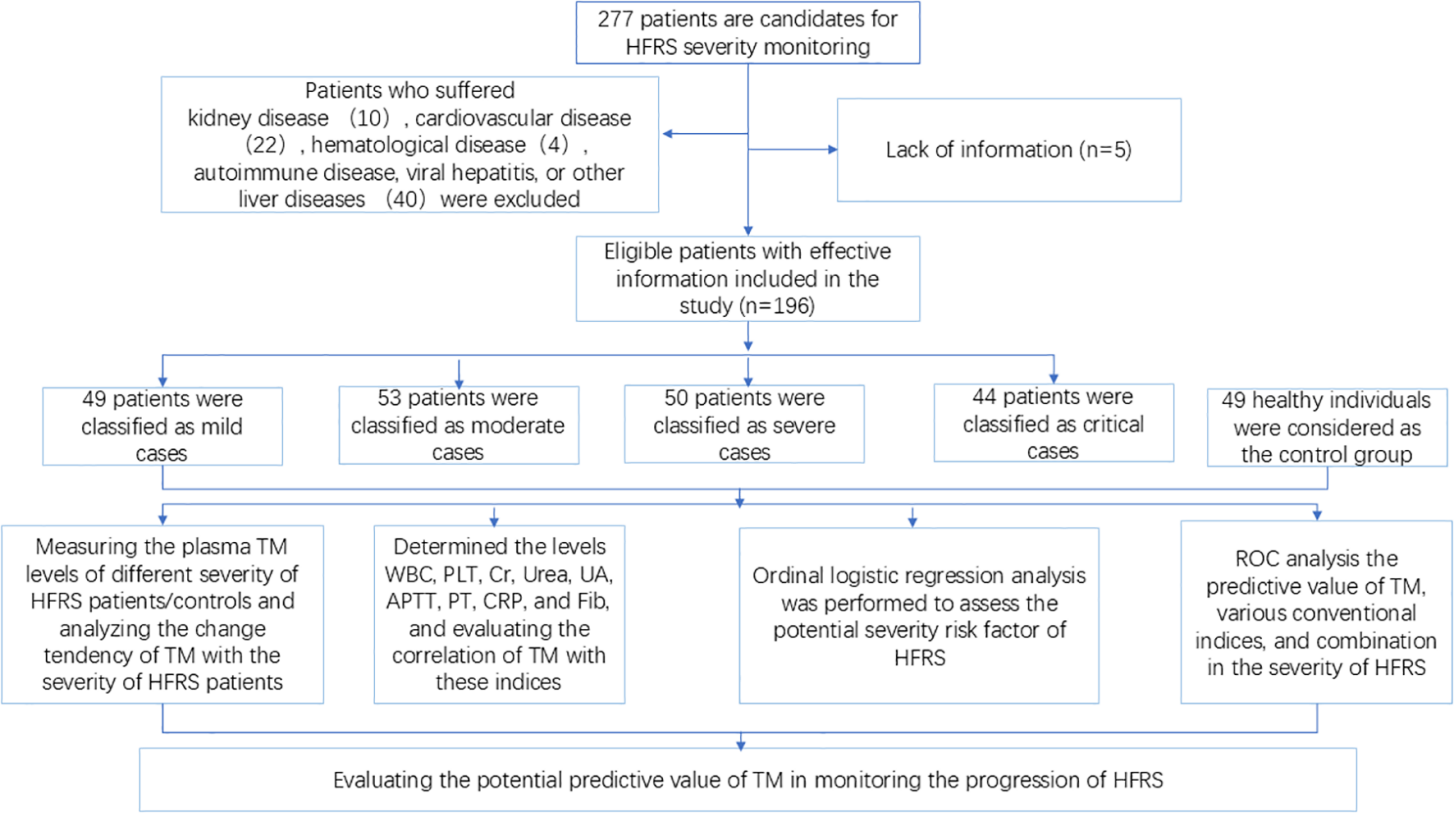
Flowchart of the study. *TM*, thrombomodulin; *HFRS*, hemorrhagic fever with renal syndrome; *ROC*, receiver operating characteristics curve.

Initially, 277 patients with laboratory-confirmed Hantaan infection were candidates for this study. However, 81 patients were excluded on the basis of the presence of kidney, cardiovascular, hematological, autoimmune, viral hepatitis, or other liver diseases. Consequently, 196 HFRS patients who were hospitalized from September 2022 to March 2024 and met the aforementioned recruitment criteria were enrolled in this study (**Figure 1**). The distribution of cases according to the classification criteria of HFRS was as follows: 49 cases were diagnosed as mild, 53 cases as moderate, 50 cases as severe, and 44 cases as critical, coupled with a gender distribution of female (14.29%, 15.09%, 14%, 15.91%) and male (85.71%, 84.91%, 86%, 84.09%), respectively (**Table 1**). The data shows that the hospitalization duration increased with the deterioration of the disease, and the peak value was found in the critical-type patients. A comparable trend was observed in conventional laboratory parameters including CRP, APTT, Cr, Urea, and UA. However, the levels of PLT declined with the progression of the disorder, contrasting with the indices mentioned above. Additionally, there was no significant change in the levels of PT, and a dramatic decline was found in the Fib levels of the critical group, However, the Fib levels in the other group did not elicit a clear change (**Table 1**).

**Table 1:** Characteristics of patients with hemorrhagic fever with renal syndrome.

	Mild Group	Moderate Group	Severe Group	Critical Group	Controls Group	Normal range
	(n=49)	(n=53)	(n=50)	(n=44)	(n=49)	
Gender						
Female, n (%)	7 (14.29)	8 (15.09)	7 (14)	7 (15.91)	10 (20.41)	–
Male, n (%)	42(85.71)	45(84.91)	43 (86)	37 (84.09)	39 (79.59)	–
Age, y ^a^	31 ± 13	40 ± 19.5	41.5 ± 15.5	44.5 ± 19.5	31 ± 11	–
Hospital stays, days ^a^	9.00 ± 4	11.00 ± 5.5	16.00 ± 6.25	19.5 ± 7.75	–	–
PLT, ×10^9^/L ^a^	103 ± 74.5	61 ± 27.5	41.5 ± 30.25	19 ± 24.5	126.5 ± 45.5	100–300
CRP, mg/L ^a^	20.01 ± 19.04	32.07 ± 34.72	43.7 ± 35.1	56.38 ± 56.97	4.56 ± 2.59	5-10
APTT, sec ^a^	33.3 ± 7.42	41.66 ± 12.43	47.01 ± 22.34	74.26 ± 44.75	26.69 ± 9.05	24 – 38
PT, sec ^a^	13.44 ± 1.90	13.43 ± 2.12	13.24 ± 2.48	14.03 ± 3.63	12.76 ± 1.94	10 – 15
Fib, g/L ^a^	3.06 ± 0.91	3.08 ± 1.05	3.04 ± 0.96	1.94 ± 0.9	2.44 ± 1.01	2 – 4
Cr, μmol/L ^a^	97.6 ± 57.8	227.6 ± 171.8	434.55 ± 238.28	403 ± 271.9	94.6 ± 23.45	70 – 115
Urea, mmol/L ^a^	6.43 ± 3.33	12.00 ± 7.77	19.75 ± 9.2	21.16 ± 12.17	4.02 ± 1.58	1.7 – 8.3
UA, μmol/L ^a^	378.64 ± 161.9	474.8 ± 83.35	527.9 ± 126.05	537 ± 236.75	237 ± 110.3	202– 416

*PLT*, Platelets; *CRP*, C-reactive protein; *APTT*, Activated partial thromboplastin time; *PT*, Prothrombin time; *Fib*, Fibrinogen; *Cr*, Creatinine; *UA*, Uric acid.

^a^Data are presented as Medians ± IQR (interquartile ranges).

### 3.2 Correlation of the levels of TM with the severity of HFRS

**Figure 2:**
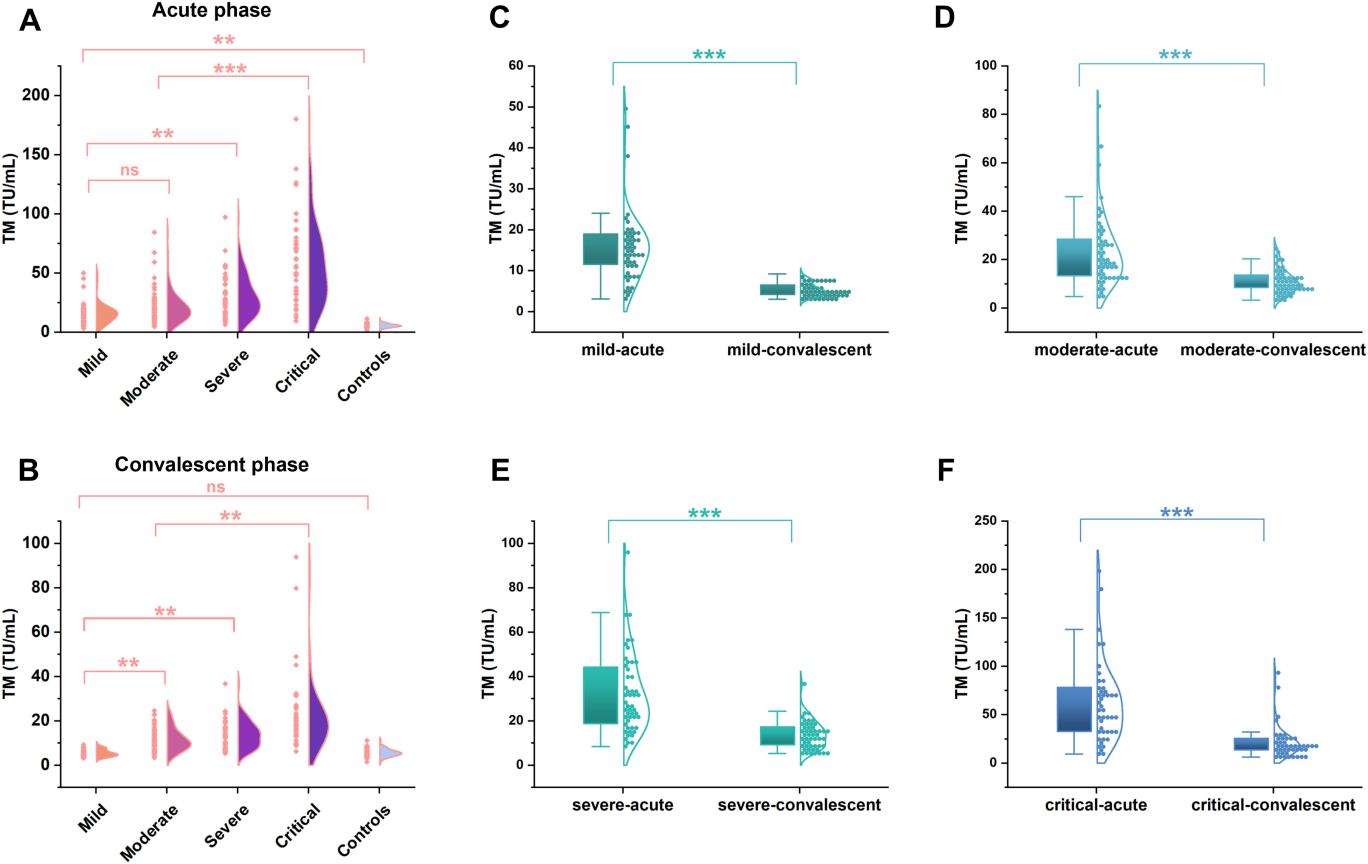
The TM levels in patients with hemorrhagic fever with renal syndrome of varying severity. The concentrations of TM in different severity of HFRS patients (n=196) were determined by the chemiluminescence immunoassay. A significant increase was found from the mild to critical type patients (**p <0.01, **A**) at the acute phase, even though a significant difference was not observed between the mild and moderate type patients (ns; p >0.05, **A**). While a remarkable decline in TM levels emerged from the acute phase to the convalescent phase in each patient group (***p <0.001, **C–F**), but the TM levels in the convalescent phase were still higher than that of the controls (**p <0.01, **B**), except a similar levels between the mild type patients and the controls (ns; p >0.05, **B**).

The levels of TM expressions were measured in the subgroups to investigate the correlation between this molecule and the progression of HFRS. As a result, the levels of TM showed an increasing tendency with the aggravation of the disorder, and the peak value was presented in the critical group in the acute phase (**Figure 2A**); in particular, a significant change can be found in the mild type patients compared to the controls, despite it being in the early stage of the HFRS (**Figure 2A**; *p*< 0.01). Subsequently, dramatic declines in TM levels were observed from the acute to the convalescent phases in each patient group category (**Figures 2C–F**; *p*< 0.001). Nonetheless, the TM levels in the critical-type patients in the convalescent phase were still higher than those of other patient types in the same phase (**Figure 2B**; *p*< 0.01), even though a clear difference was not found between the mild-type patients and the controls in the convalescent phase (**Figure 2B**; *p*> 0.05).

### 3.3 Correlation of the TM with the conventional parameters

**Figure 3:**
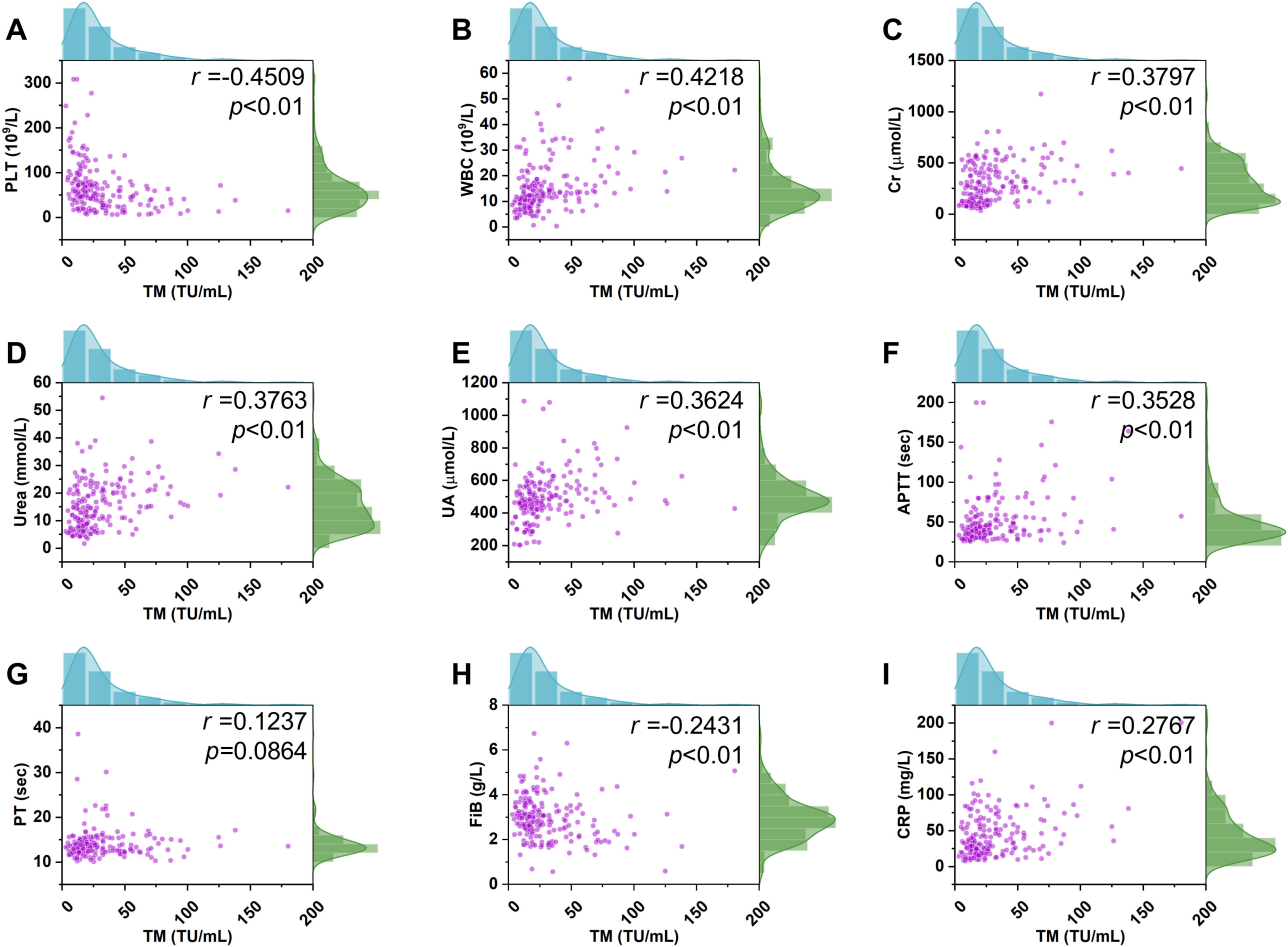
Correlation of the TM with the conventional parameters. A positive correlation between TM and the WBC levels in HFRS patients (n=196) was observed (p <0.01, r= 0.4218, **B**). A similar positive relationship between TM levels and other conventional indices including Cr, urea, UA, APTT, and CRP was also noted (p <0.01, r= 0.3797, p <0.01, r= 0.3763, p <0.01, r= 0.3624, p <0.01, r= 0.3528, p <0.01, r= 0.2767, **C–F**, **I**). In contrast, a negative correlation of TM levels with PLT and Fib was found (p <0.01, r= -0.4509, p <0.01, r= 0.2431, **A, H**). However, a significant correlation of TM levels with PT was not observed (p =0.0864, r= 0.1237, **G**).

Given the increased permeability of the microvascular beds of affected organs composed of endothelial cells, such as the kidney has been reported in HFRS patients, and coagulation disorder can also be found in such patients, the relationship between TM and various related conventional parameters, including PLT, Cr, urea, UA, APTT, PT, Fib, WBC, and CRP, was evaluated. Consequently, spearman correlation analysis showed that TM levels were negatively associated with PLT levels (**Figure 3A**; *p*< 0.01, *r*= -0.4509), and a similar tendency was also found between the TM and Fib levels (**Figure 3H**; *p*< 0.01, *r*= -0.2431). Conversely, fluctuation of the TM levels was positively correlated with the change in WBC levels (**Figure 3B**; *p*< 0.01, *r*= 0.4218), and a similar phenomenon between the TM, Cr, urea, UA, APTT, and CRP was also noted (**Figures 3C–F, I**; *p*< 0.01). Meanwhile, the association between TM levels and these parameters varied, including the strongest association between the WBC and TM; a relatively weaker correlation between the Cr, urea, UA, and TM; and a minimal correlation between CRP and TM. In addition, no significant correlation was found between PT and TM levels (**Figure 3G**; *p*=0.0864).

### 3.4 Ordinal logistic regression analysis for the severity risk factor in HFRS patients

Ordinal logistic regression analysis showed that TM, WBC, PLT, Cr, Urea, UA, and CRP levels were correlated with the severity of HFRS. Of note, the tendency of these indices varied, presenting as elevated TM, WBC, Cr, Urea, UA, and CRP levels associated with the progression of HFRS. In contrast, the decline in PLT levels correlated with the progression of this disorder (**Table 2**).

**Table 2:** Ordinal logistic regression analysis for severity risk factor in HFRS patients.

Variables	Estimated value	OR (95% *CI*)	*P-*value
TM	0.038	1.039 (0.017-0.059)	<0.001
WBC	0.076	1.079 (0.026-0.126)	0.003
PLT	-0.028	0.972(-0.01- -0.016)	<0.001
Cr	0.002	1.002(3.739E-05-0.005)	0.047
Urea	0.085	1.088(0.026-0.143)	0.004
UA	0.007	1.007(0.003-0.01)	<0.001
APTT	-0.006	0.994(-0.02-0.009)	0.445
PT	0.085	1.089(-0.025-0.196)	0.130
CRP	0.014	1.014(0.003-0.024)	0.012
Fib	-0.057	0.945(-0.484-0.371)	0.796

*TM*, Thrombomodulin; *WBC*, White blood cells; *PLT*, Platelets; *Cr*, Creatinine; *UA*, Uric acid; *APTT*, Activated partial thromboplastin time; *PT*, Prothrombin time; *CRP*, C-reactive protein; *Fib*, Fibrinogen.

*OR* The OR value reflects the relative risk of an outcome occurring in the presence or absence of exposure, *CI* Confidence interval.

### 3.5 ROC curve for the predictive efficacy of HFRS severity

**Figure 4:**
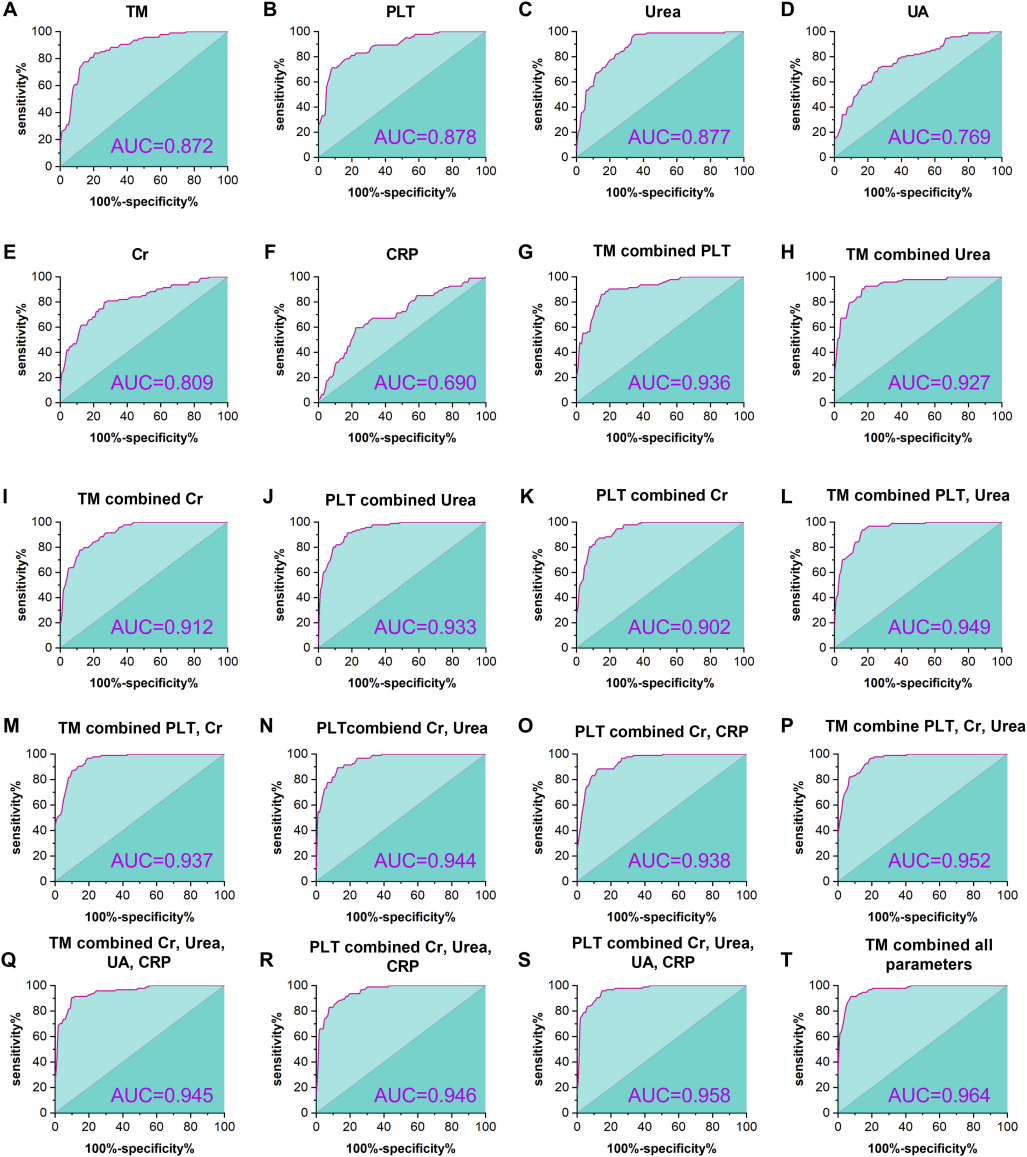
ROC curves for the predictive efficacy of HFRS severity. The figure shows the predictive efficacy of TM, PLT, urea, UA, Cr, and CRP and the combination of varying indices for the severity in HFRS patients. TM showed a significant predictive capacity, with an AUC of 0.872 (*p <*0.001, **(A)** higher than that of the conventional parameters **(D-F)**, except for the PLT and urea **(B, C)**. In addition, predictive capacity is elevated with the number of combination parameters **(G-S)**, and the most powerful prediction can be found in the combination of TM with all conventional parameters presented herein **(T)**.

ROC curves were adopted to assess the predictive capacity of TM and several conventional laboratory indices, including PLT, urea, UA, Cr, and CRP for HFRS severity. As presented in **Figure 4** and **Table 3**. ROC curve analysis demonstrated a high predictive value of TM for HFRS severity, with an AUC of 0.872 (95%; *CI*: 0.822 – 0.921, *p*<0.001), while PLT and urea elicited a similar predictive capacity with AUC of 0.878 (95%; *CI*: 0.830 – 0.925, *p*<0.001) and 0.877 (95%; *CI*: 0.829 – 0.925, *p*<0.001), respectively. Nonetheless, the latter parameters possessed a weaker balance between sensitivity and specificity compared with the TM, and the red blood fragments seriously interfered with the determination of these two indices in the clinical laboratory, which resulted in confusion and misdiagnosis and posed a challenge for clinicians. Furthermore, combining TM with one or more conventional parameters presented a higher predictive capacity for HFRS severity than single indices. The combination of TM with PLT exhibited a better predictive property than that of TM combined with renal function parameters such as urea or UA. Notably, although AUC in the pattern of TM combined with Cr, Urea, UA, and CRP (AUC: 0.945; 95%; *CI*: 0.914 – 0.975, *p*<0.001) is slightly smaller than that of PLT combined with the same parameters (AUC: 0.958; 95%; *CI*: 0.932 – 0.985, *p*<0.001), the former combination pattern presented a better balance between the sensitivity and specificity. Meanwhile, TM combined with all conventional parameters presented herein shows an impressive predictive capacity for HFRS severity with an AUC of 0.964, sensitivity of 91.49%, and specificity of 92.16%, respectively.

**Table 3:** Predictive efficacy of TM and conventional laboratory indices, and the combination of various indices.

Variables	AUC (95% *CI*)	Sensitivity (%)	Specificity (%)	*P-*value
TM	0.872 (0.822-0.921)	84.04	79.41	<0.001
PLT	0.878 (0.830-0.925)	70.21	92.16	<0.001
Urea	0.877 (0.829-0.925)	97.87	64.71	<0.001
UA	0.769 (0.703-0.834)	71.28	73.53	<0.001
Cr	0.809 (0.748-0.870)	79.79	73.53	<0.001
CRP	0.690 (0.615-0.764)	59.57	77.45	<0.001
TM combined PLT	0.936 (0.904-0.968)	87.20	86.30	<0.001
TM combined Urea	0.927 (0.891-0.963)	92.55	81.37	<0.001
TM combined Cr	0.912 (0.874-0.950)	77.66	88.24	<0.001
PLT combined Urea	0.933 (0.898-0.967)	91.50	82.40	<0.001
PLT combined Cr	0.902 (0.860-0.945)	90.43	80.39	<0.001
TM combined PLT, and Urea	0.949 (0.922-0.977)	96.81	80.39	<0.001
TM combined PLT, and Cr	0.937 (0.904-0.970)	93.62	83.33	<0.001
PLT combined Cr, and Urea	0.944 (0.914-0.974)	89.40	87.30	<0.001
PLT combined Cr, and CRP	0.938 (0.906-0.970)	88.30	87.30	<0.001
TM combined PLT, Cr, and Urea	0.952(0.926-0.979)	96.80	80.40	<0.001
TM combined Cr, Urea, UA, CRP	0.945(0.914-0.975)	90.40	90.20	<0.001
PLT combined Cr, Urea, and CRP	0.946 (0.916-0.975)	82.90	92.20	<0.001
PLT combined Cr, Urea, UA, CRP	0.958 (0.932-0.985)	95.70	85.30	<0.001
TM combined all parameters	0.964 (0.941-0.987)	91.49	92.16	<0.001

*AUC*, Area under the ROC curve; *CI*, Confidence interval; *TM*, Thrombomodulin; *PLT*, Platelets; *UA*, Uric acid; *Cr*, Creatinine; *CRP*, C-reactive protein.

## 4 Discussion

HFRS, which is known as a zoonotic disease, has attracted increasing attention for its profound influence on public health, the healthcare system, and socioeconomic status, even though the causative agent discovery of this disorder dates back to 1978 (Lee et al., 1978). Although supportive treatment, as well as vaccination, made a great contribution to the alleviation and prevention of this disorder (Dheerasekara et al., 2020), owing to the lack of a reliable indicator to reflect the severity of HFRS in a timely manner, it still poses a challenge for clinicians to monitor and manage the condition of HFRS patients effectively.

In this study, we investigated the levels of TM in HFRS patients of varying severities and found that the TM expression increased significantly in samples from mild to critical-type patients, meanwhile, the levels of this molecule in the acute phase of HFRS patients were markedly higher than those of convalescent-phase in each severity type. It is widely accepted that endothelial cells are the primary targets of HTNV infection, additionally, TM is considered to play a central role in the biological function of various organs, as it can be expressed by endothelial cells that line all vessels (Kanerva et al., 1998; Maruyama et al., 1985). Thus, the tendency of TM levels to change, which is observed herein, further confirms the close relationship between TM and endothelial cells and suggests the potential role of this molecule in monitoring the progression of HFRS caused by HTNV. Furthermore, it has been documented that immune-mediated mechanisms are involved in HFRS pathogenesis and the onset of HFRS may be related to immune dysfunction and pathological damage caused by disorders of immune regulation (Huang et al., 1994). Noteworthy, TM is well-positioned to participate in the regulation of innate immunity, and the serine-threonine-rich region of this molecule can bind to adhesion molecules such as Leukocyte Function-associated Antigen-1 (LFA-1), and the latter plays a key role in T cell priming, differentiation, and effector functions (Weiler and Isermann, 2003; Loghmani and Conway, 2018; Gerard et al., 2021). In addition, it has been revealed that HTNV infection induces a strong cellular immune response, and T cells, particularly CD8 T cells, play a vital role in eliminating HTNV (Huang et al., 1994; Xie et al., 2013), therefore, the fluctuation of TM levels in HFRS patients seems also to reflect both the innate and cellular immune response of patients to some extent. Likewise, capillary leak syndrome with hemoconcentration induced by increased permeability is one major manifestation of HFRS that is closely related to the intercellular adhesion molecule-1 (ICAM-1) (Terajima and Ennis, 2011; Yu et al., 2014), meanwhile, this molecule can cooperate with LFA-1 which is further responsible for the migration of lymphocytes from the blood vessels and peripheral tissues into the lymph node coupled with the interaction between the TM and LFA-1 mentioned above (Guo et al., 2023). Thus, elevated levels of TM in patients with mild to severe HFRS may, on the other hand, indicate the degree of increased vascular permeability in the patient.

Renal injury or failure is another major manifestation of HFRS, and Cr, UA, and urea levels are important parameters that reflect the degree of kidney injury (Jonsson et al., 2010). This study determined the levels of these indices, and the correlation of TM with these indices was also analyzed. The data shows that the TM levels of HFRS patients were positively correlated with increases in Cr, UA, and urea, indicating that overexpression of TM may indirectly reflect the impairment of vascular endothelial cells and worsening of renal injury during the deterioration of patients with HFRS. In addition, we examined the correlation between TM and PLT levels, given that thrombocytopenia is a hallmark of HFRS (Muranyi et al., 2005), Our findings revealed an increasing tendency of TM levels along with the progression of HFRS. Consequently, a negative correlation between TM and PLT was identified, in contrast to the relationship observed between TM, Cr, UA, and urea. Given that PLT exhibits hallmark structural and functional characteristics that are associated with their participation in host defense against infection, such as rapid response to agonists associated with vascular trauma or infection and accumulation at sites of endovascular damage or microbial colonization, and it was found that activation of PLT by agonists generated in situations aforementioned could enhance PLT interactions with complement proteins, humoral immune components, leukocytes, and endothelial cells (Yeaman, 1997). Likewise, PLT also possesses the property of binding, aggregating, and internalizing microorganisms, which could contribute to the clearance of pathogens from the bloodstream (Clawson and White, 1971). These findings suggest the important role of PLT in the pathogenesis of HFRS, coupled with the close relationship between PLT and TM, which indirectly implies a valuable role of TM in the course of HFRS. Furthermore, considering that coagulation disorder can also be observed in HFRS patients, and refractory shock, hemorrhage-related complications, disseminated intravascular coagulation (DIC), and multiple organ dysfunction syndrome (MODS) result in the death of severe HFRS patients (Chen et al., 2024; Lee et al., 1983). The correlation of TM with coagulation parameters involving the APTT, PT, and Fib was analyzed. It was found that the APTT levels positively correlated with the TM levels, while a converse relationship between the Fib and TM was observed, which was consistent with the previous studies and further indicated the close relationship between TM and the progression of HFRS (Chen et al., 2024). Noteworthy, a significant correlation between TM and PT was not found, given the complexity of coagulation and the corresponding part in the mechanism of HFRS is yet unclear, the relationship between these two molecules remains to be elucidated.

As reported, the pathophysiology of organ damage that emerged in the HTNV infection also motivates the increase in inflammatory biomarkers, including the C-reactive protein (CRP), coupled with the sensitivity of this parameter for viral infection and inflammation, it was used to evaluate disease severity and predict outcomes of COVID-19 patients and play a vital role in the treatment of HFRS (Che et al., 2022; Shin et al., 2018; Liu et al., 2020). Thus, the correlation of TM with CRP, and the conventional inflammation parameter, white blood cells, was evaluated herein. A positive relationship was found between TM, WBC, and CRP. Of note, the correlation of TM with WBC was closer than that of TM with CRP, which suggests that the component variety of WBC, including the lymphocytes, and monocytes, may contribute to this phenomenon, considering the reports that these leukocytes participate in the mechanism of HFRS, and this further suggested the close relationship between the TM levels change and the course of HFRS (Xie et al., 2009; Markotic et al., 2007).

The complexity and heterogeneity of the clinical course and prognosis of patients with HFRS must be seriously considered, and a reliable parameter that predicts the severity or progression of HFRS would be beneficial for effective patient treatment and management. ROC analysis showed that TM had a statistically significant value for the severity of HFRS, with a better balance between sensitivity and specificity than other markers. Noteworthy, although PLT showed the highest sensitivity among the conventional laboratory parameters, it is less promising for clinical use as PLT counts are subject to interference from red blood cell fragments and hemolysis in clinical laboratory examination. Meanwhile, hemolysis is also the most frequent interference affecting clinical chemistry parameters, including UA and urea, in clinical laboratories (Perovic and Dolcic, 2019). Thus, TM levels in combination with multiple conventional parameters could be a reliable method to guide clinical treatment. Indeed, the results herein demonstrated that the predictive property increased with the combination number of indices. And the renal parameters such as urea or UA and Cr combined with TM or PLT show a better predictive capacity than that of CRP combined with these renal parameters. Furthermore, TM combined with Cr, urea, UA, and CRP presented a better sensitivity and specificity than that of PLT combined with these indices. which further reflects the significant role of TM in predicting the severity of HFRS caused by HTNV.

Admittedly, a meaningful predictive value of TM on the severity and course of HFRS was observed in this study; however, considering the nature of a prospective study herein, the limitations should not be ignored. Specifically, this study was conducted in a single center for infectious diseases with a limited magnitude of study population, which may weaken the prediction power, thus, it is warranted to enroll more cases within a multicenter to broaden the generalizability of the findings in the coming days. Furthermore, owing to the objective law of disorder development, admission procedures, varying conditions of patients, and segments involving the sample collection, transportation, registration, and examination, all of which should be unified in future studies. Additionally, the limitations including experimental measurement errors, human errors, and the clinician’s non-comprehensive understanding of the guidelines for HFRS treatment leading to the information of target parameters missed, should not be obscured.

## 5 Conclusions

Overall, our findings showed that elevated plasma levels of TM in HFRS patients were correlated with increasing levels of Cr, urea, and UA, which indicated TM levels were closely associated with the severity of HFRS, and was further demonstrated by the ROC curve analysis, clearly showed the predictive value of TM for the seriousness of HFRS. The detection of plasma TM levels, in particular, the combination determination of TM with multiple conventional parameters would benefit clinicians in effectively monitoring the conditions of HFRS patients, and further studies in the coming days focusing on the role of TM in the course of HFRS from various perspectives would better reveal the value of this molecule as well as contribute to the management of this population to improve the therapeutic effect and prognosis of HFRS.

## Data Availability

The raw data supporting the conclusions of this article will be made available by the authors, without undue reservation.
